# Subjective cognitive concerns, *APOE* ε4, PTSD symptoms, and risk for dementia among older veterans

**DOI:** 10.1186/s13195-024-01512-w

**Published:** 2024-06-29

**Authors:** Zoe E. Neale, Jennifer R. Fonda, Mark W. Miller, Erika J. Wolf, Rui Zhang, Richard Sherva, Kelly M. Harrington, Victoria Merritt, Matthew S. Panizzon, Richard L. Hauger, J. Michael Gaziano, the VA Million Veteran Program, Sumitra Muralidhar, Jennifer Moser, Jennifer E. Deen, Philip S. Tsao, Sumitra Muralidhar, Elizabeth Hauser, Amy Kilbourne, Shiuh-Wen Luoh, Michael Matheny, Dave Oslin, Philip S. Tsao, Lori Churby, Stacey B. Whitbourne, Jessica V. Brewer, Shahpoor Alex Shayan, Luis E. Selva, Saiju Pyarajan, Kelly Cho, Scott L. DuVall, Mary T. Brophy, Philip S. Tsao, Brady Stephens, Dean P. Argyres, Themistocles L. Assimes, Adriana Hung, Henry Kranzler, Samuel Aguayo, Sunil Ahuja, Kathrina Alexander, Xiao M. Androulakis, Prakash Balasubramanian, Zuhair Ballas, Jean Beckham, Sujata Bhushan, Edward Boyko, David Cohen, Louis Dellitalia, L. Christine Faulk, Joseph Fayad, Daryl Fujii, Saib Gappy, Frank Gesek, Jennifer Greco, Michael Godschalk, Todd W. Gress, Samir Gupta, Salvador Gutierrez, John Harley, Kimberly Hammer, Mark Hamner, Adriana Hung, Robin Hurley, Pran Iruvanti, Frank Jacono, Darshana Jhala, Scott Kinlay, Jon Klein, Michael Landry, Peter Liang, Suthat Liangpunsakul, Jack Lichy, C. Scott Mahan, Ronnie Marrache, Stephen Mastorides, Elisabeth Mates, Kristin Mattocks, Paul Meyer, Jonathan Moorman, Timothy Morgan, Maureen Murdoch, James Norton, Olaoluwa Okusaga, Kris Ann Oursler, Ana Palacio, Samuel Poon, Emily Potter, Michael Rauchman, Richard Servatius, Satish Sharma, River Smith, Peruvemba Sriram, Patrick Strollo, Neeraj Tandon, Philip Tsao, Gerardo Villareal, Agnes Wallbom, Jessica Walsh, John Wells, Jeffrey Whittle, Mary Whooley, Allison E. Williams, Peter Wilson, Junzhe Xu, Shing Shing Yeh, Mark W. Logue

**Affiliations:** 1 National Center for PTSD, Behavioral Sciences Division, VA Boston Healthcare System, 150 South Huntington Ave (116B-2), Boston, MA 02130 USA; 2 Department of Psychiatry and Behavioral Sciences, SUNY Downstate Health Sciences University, Brooklyn, NY USA; 3 Institute for Genomics in Health (IGH), SUNY Downstate Health Sciences University, Brooklyn, NY USA; 4 Department of Psychiatry, Boston University Chobanian & Avedisian School of Medicine, Boston, MA 02130 USA; 5 Translational Research Center for TBI and Stress Disorders (TRACTS) and Geriatric Research, Educational and Clinical Center (GRECC), VA Boston Healthcare System, Boston, MA USA; 6 Department of Psychiatry, Harvard Medical School, Boston, MA USA; 7 Biomedical Genetics, Boston University Chobanian & Avedisian School of Medicine, Boston, MA 02130 USA; 8 Department of Biostatistics, Boston University School of Public Health, Boston, MA USA; 9 Million Veteran Program (MVP) Coordinating Center, VA Boston Healthcare System, Boston, MA USA; 10 Center of Excellence for Stress and Mental Health, VA San Diego Healthcare System, San Diego, CA USA; 11 Department of Psychiatry, University of California, La Jolla, San Diego, CA USA; 12 Center for Behavior Genetics of Aging, University of California, La Jolla, San Diego, CA USA; 13 Division of Aging, Brigham & Women’s Hospital, Harvard Medical School, Boston, MA USA

**Keywords:** Dementia, *APOE ε4*, TBI, PTSD, Survival analysis

## Abstract

**Background:**

Posttraumatic stress disorder (PTSD) and traumatic brain injury (TBI) are associated with self-reported problems with cognition as well as risk for Alzheimer’s disease and related dementias (ADRD). Overlapping symptom profiles observed in cognitive disorders, psychiatric disorders, and environmental exposures (e.g., head injury) can complicate the detection of early signs of ADRD. The interplay between PTSD, head injury, subjective (self-reported) cognitive concerns and genetic risk for ADRD is also not well understood, particularly in diverse ancestry groups.

**Methods:**

Using data from the U.S. Department of Veterans Affairs (VA) Million Veteran Program (MVP), we examined the relationship between dementia risk factors (*APOE ε4*, PTSD, TBI) and subjective cognitive concerns (SCC) measured in individuals of European (*n* = 140,921), African (*n* = 15,788), and Hispanic (*n* = 8,064) ancestry (EA, AA, and HA, respectively). We then used data from the VA electronic medical record to perform a retrospective survival analysis evaluating PTSD, TBI, *APOE ε4,* and SCC and their associations with risk of conversion to ADRD in Veterans aged 65 and older.

**Results:**

PTSD symptoms (B = 0.50–0.52, *p* < 1E-250) and probable TBI (B = 0.05–0.19, *p* = 1.51E-07 – 0.002) were positively associated with SCC across all three ancestry groups. *APOE ε4* was associated with greater SCC in EA Veterans aged 65 and older (B = 0.037, *p* = 1.88E-12). Results of Cox models indicated that PTSD symptoms (hazard ratio [HR] = 1.13–1.21), *APOE ε4* (HR = 1.73–2.05) and SCC (HR = 1.18–1.37) were positively associated with risk for ADRD across all three ancestry groups. In the EA group, probable TBI also contributed to increased risk of ADRD (HR = 1.18).

**Conclusions:**

The findings underscore the value of SCC as an indicator of ADRD risk in Veterans 65 and older when considered in conjunction with other influential genetic, clinical, and demographic risk factors.

**Supplementary Information:**

The online version contains supplementary material available at 10.1186/s13195-024-01512-w.

## Introduction

Dementia is a debilitating condition affecting approximately 10% of Americans over the age of 65 [45]. The estimated prevalence of dementia in Veterans receiving treatment at the US Department of Veterans Affairs (VA) medical centers is similar (9–10%), though expected to rise dramatically in coming years as the Veteran population ages [74]. Posttraumatic stress disorder (PTSD), traumatic brain injury (TBI), and depression are more prevalent among Veterans relative to the general population [79] and also confer risk for dementia, further suggesting the importance of studying dementia among Veterans. Studies of the genetics of Alzheimer’s disease (AD), the most common form of dementia, have identified multiple AD-risk associated loci [12], the strongest of which is the apolipoprotein E ε4 (*APOE* ε4*)* variant [62]. The prevalence of and AD risk conferred by *APOE* ε4 varies by genetic ancestry. For example, the risk of AD is higher in African American and Hispanic/Latino populations than in European-ancestry populations [38, 46, 55]. *APOE* ε4 is also more prevalent in these populations, but the *APOE* ε4 effect on AD risk is less than it is for those of European ancestry [19, 41, 70, 71]. Racial/ethnic minority groups have also been underrepresented in ADRD neurobiological and genomic research [9, 40, 50, 58], thereby limiting understanding of *APOE* ε4 and its interaction with other influential risk factors in these groups. *APOE* ε4 may increase risk for AD via its associations with environmental and behavioral factors that confer risk for dementia, such as stress exposure, sedentary lifestyle, trauma exposure, and TBI [17, 26, 42, 44, 80].

It is critical to identify the early signs of dementia as this may help slow the disease, reduce disease burden, and contribute to the development of new treatments for ADRD [28, 56, 57]. One early indicator of dementia risk is the self-perception of difficulties in memory, attention, concentration, or executive functioning, which is referred to as subjective cognitive concerns (SCC). A related concept is the perception that cognitive performance in these realms has declined from some previous level, which is known as subjective cognitive decline (SCD). SCD is one of the earliest reported symptoms of AD [29, 30]. Various studies have shown SCC and SCD to be predictive of subsequent objectively measured memory decline [37, 39, 59], increased risk for ADRD (see [48] for a review), and biological markers of AD risk including *APOE* ε4 (see [3] for review), levels of amyloid beta in cerebrospinal fluid (e.g., [4, 35, 72]), and brain morphology (e.g., [49, 53, 61]). However, the use of SCC as an early indicator of dementia is complicated by the fact that it also reflects the manifestations of various psychiatric conditions [76]. Many studies have also demonstrated a strong association between PTSD and SCC [16, 25, 47, 52, 66]. One study of September 11, 2001 World Trade Center (WTC) first responders with a mean age 45.9 years at baseline showed that the association between intensity of WTC exposure and later SCC was almost entirely mediated by mental health comorbidities, with PTSD having the largest impact [68]. Therefore, it is important to take psychiatric factors into consideration in analyses of the relationship between SCC and risk for dementia.

We undertook this study to clarify the relationships between SCC and psychiatric and genetic risk factors for ADRD to advance our understanding of how these associations vary across age and race/ethnicity using data from the U.S. Department of Veterans Affairs’ Million Veteran Project (MVP). MVP is one of the world’s largest and most diverse cohort studies of the genetics of human disease and traits inclusive of over a million enrolled U.S. Veterans. Our first aim was to examine if previously identified associations between genetic and psychiatric risk factors for ADRD replicated when considering SCC rather than objective determinations of ADRD. We examined these associations in Veterans of European ancestry (EA); African ancestry (AA); and Hispanic ancestry (HA) to capture differential *APOE* ε4 effects and to examine the possibility of differential impact of exposures by ancestry. We hypothesized that SCC would be associated with *APOE* ε4 in older Veterans, even after accounting for demographic and psychiatric dementia risk factors, and that the magnitude of this association would differ by ancestry. Next, capitalizing on the availability of longitudinal electronic medical record (EMR) data for MVP participants, our second aim was to evaluate the prognostic value of SCC as an indicator of future ADRD diagnoses in the medical chart. To do so, we conducted a retrospective cohort survival analysis using Cox regression models to evaluate the associations between PTSD, TBI, *APOE* ε4*,* and SCC on risk for dementia onset as determined in the medical record among Veterans aged 65 and older. This represents an important extension of our previous MVP study that examined gene-by-environment interaction (GxE) effects of PTSD, TBI, and *APOE* ε4 on ADRD risk in Veterans 65 and older using a cross-sectional (case–control) framework: that study found that the association between PTSD and TBI with ADRD was stronger as a function of *APOE* ε4 [42]. Here, we hypothesized that increased SCC would be associated with higher risk for ADRD onset after accounting for genetic risk (*APOE* ε4), PTSD, TBI, demographic, and lifestyle covariates.

## Methods

### Participants and procedures

MVP is a national research program aimed at improving Veteran health by examining the impact of genetics, lifestyle, and military experiences on health outcomes [20]. Participants take part in surveys, provide blood samples, and consent to access of their VA EMR. Here, we utilized data from the MVP 20.1 phenotype release, the Phase 3 genotype release, and the MVP Baseline Survey and Lifestyle Survey [51]. We excluded participants with a history of schizophrenia and bipolar disorder based on either self-report in the MVP Baseline Survey or presence of International Classification of Diseases (ICD) codes for schizophrenia or bipolar disorder (ICD-9: 296.4, 296.5, 296.6, 296.7, 296.8; ICD-10: F20, F25.9, F31) in the EMR. Veterans with ADRD or mild cognitive impairment (MCI) codes predating the Lifestyle Survey and those with other non-ADRD dementia codes were excluded (See Supplementary Table 1). Ancestry was identified using the genotype-informed Harmonized Ancestry and Race/Ethnicity (HARE) method [18]. HARE classification is very similar to genotype-based clustering, except where there is a mismatch between the self-reported ancestry and genetic clustering, in which case, subjects were not assigned to a group. The final analytic sample included 166,347 participants aged 45 and older who had genetic data, provided responses to MVP Baseline and Lifestyle Surveys, and were classified within the three largest ancestry groups in MVP: EA (*N* = 143,298), AA (*N* = 16,250), and HA (*N* = 6,799). We further divided these groups into three age cohorts based on the age at completion of the Lifestyle Survey: early middle age (45–54), presumably before the age of substantial AD-associated neurological changes; late middle age (age 55–64), when presumably AD-associated neurocognitive changes would be more apparent, but prior to the typical age of AD risk; and older age (65 +) at which time Veterans would be at risk for dementia onset. The Cox regression models predicting time to ADRD diagnosis were conducted only in the age 65 and older age cohorts as the younger ADRD cases may represent the distinct “early-onset” form of AD.

### Measures

#### Subjective cognitive concerns

SCC scores were calculated based on six items administered to MVP participants in the MVP Lifestyle Survey derived from the Medical Outcomes Study Cognitive Functioning Scale (MOS-Cog-R; [77]), a revised version of the MOS Cognitive functioning scale that has been used in more than 20 studies, including two clinical trials [21, 67]. Items in the MOS-Cog-R assess how much a respondent has experienced difficulty in the last month with six different cognitive tasks associated with memory, attention, concentration, problem-solving, and confusion. For example, “*How many times in the last month…did you have difficulty reasoning and solving problems (e.g., making plans, making decisions, learning new things)?*” and “*How many times in the last month…did you forget (e.g., things that happened recently, where you put things, appointments)?*” The response options are presented on a Likert-like scale ranging from (0) “*Never*,” to (5) “*All of the time*.” Items were coded (0–5) such that higher scores indicated more problems with cognition and were then summed on a total scale ranging from 0–30. In preparation for this study, we conducted a factor analysis and found that the six MOS-Cog-R items loaded onto a single factor; thus, we used a sum score of all items (standard for the MOS-Cog-R) rather than examining cognitive domains separately.

#### PTSD and depression/anxiety symptoms

PTSD symptoms were assessed in the MVP Lifestyle Survey using the 17-item version of the PTSD Checklist (PCL; [73]) based on the Diagnostic and Statistical Manual of Mental Disorders, 4th edition criteria [5]. Participants were asked to indicate how much they have been bothered by symptoms related to stressful experiences over the past 30 days. Response options ranged from (1) *Not at all* to (5) *Extremely*, with total scores ranging from 17–85. Depression and anxiety symptoms were assessed using the Patient Health Questionnaire-4 (PHQ-4), which is a 4-item self-report measure in which participants are asked to report how much they have been bothered by symptoms of depression and anxiety over the past two weeks. Response options ranged from (0) *Not at all* to (3) *Nearly every day*. Depression/anxiety symptoms and PTSD symptoms were strongly correlated (*r* = 0.77), so to avoid multicollinearity, we focused our analyses on PTSD symptoms.

#### Traumatic Brain Injury (TBI)

Self-report was used to capture TBI as historical TBI events, and in particular combat-related TBIs for Veterans in the older age group, may not be reflected in the EMR. History of probable TBI was assessed via self-report in the MVP Baseline Survey. Participants who indicated they had been diagnosed with either a “traumatic brain injury” or “concussion or loss of consciousness” were defined as a probable TBI case.

#### Alzheimer’s Disease and Related Dementias (ADRD)

Consistent with previous EMR studies of dementia [6, 15, 32, 42], our analyses focused on ADRD (rather than AD only) due to the lack of AD-specific biomarkers in the EMR that would allow for a more detailed diagnosis. As AD is the most common form of dementia, comprising 60–80% of dementia cases [1], the majority of identified ADRD cases are likely AD cases. Our ADRD diagnostic algorithm is described in detail elsewhere [42]; briefly, ADRD cases were those who had at least two ICD-9 or ICD-10 codes for AD, a related dementia such as Lewy body dementia or vascular dementia, or other non-specific dementia codes in the EMR (see Supplemental Table 1). Controls were defined as MVP participants who did not have a history of ICD codes for all-cause dementia or MCI, or prescriptions for dementia medications based on pharmacy data available in the EMR (see Supplemental Table 2 for a list of these medications).

#### APOE genotyping

Detailed information about MVP DNA sampling, genotyping, and quality control procedures is provided in Hunter-Zinck et al. [27]. Briefly, samples were genotyped using the MVP 1.0 custom Axiom array, which assessed 668,418 genetic markers. The MVP Bioinformatics core completed processing, cleaning, and imputation of the genotypic data. The *APOE* genotype was determined from the well-imputed genotypes of two single-nucleotide polymorphisms, rs7412 (imputation r^2^ = 0.99 in EA and HA cohorts, r^2^ = 0.98 in AA cohort) and rs429358 (imputation r^2^ = 0.99 in EA, AA, and HA cohorts). The “best guess” imputed values for rs7412 and rs429358 with a 90% confidence threshold were used. From the *APOE* genotypes, the number of ε4 alleles were coded (0–2) and included in the analyses as a linear term.

### Data analysis

Analyses were conducted using R (v4.0.3). All analyses were conducted stratified by ancestry to account for known differences in *APOE* ε4 effects by ancestry and accommodate potential differences in measured and unmeasured non-genetic ADRD risk factors [7]. Descriptive univariate analyses comparing key variables within ancestry across age groups were calculated using ANOVA and chi-square tests. The variables for depression/anxiety symptoms (PHQ-4), PTSD symptoms, and cognitive concerns were standardized to aid in interpretation of interactions and effect sizes. We then conducted multiple linear regressions using the R *lm()* function to estimate the associations of PTSD symptoms, probable TBI, *APOE* ε4, and interactions between PTSD symptoms, probable TBI, and *APOE* ε4 on SCC after accounting for covariates in each of the three age groups. Next, we computed the hazard ratio (HR) and 95% confidence interval (CI) to examine the associations between SCC, PTSD symptoms, probable TBI, *APOE* ε4, and their interactions on proportional risk for dementia onset in individuals 65 and older using Cox regression models through the *‘survival’* package in R. We note that the Cox regression assumption of proportional hazard, which decrees that risk for the dependent variable is constant over time, may be compromised in studies of degenerative disease, such as ADRD, where risk increases with advancing age. However, alternative approaches, such as generalized Cox regression and spline models, can be difficult to interpret, and produce coefficients that are only marginally different from those in standard Cox regression [23]. Therefore, use of Cox proportional hazards models is widespread in the AD risk literature (see e.g. [24, 43, 65]). We similarly used Cox regression models in the present study. Survival analyses require a measure of time for both ADRD cases and controls. For ADRD controls, we subtracted age at MVP Lifestyle Survey from age at last visit in the EMR for a measure of years monitored (censored). For ADRD cases, we subtracted age at MVP Lifestyle Survey from age at diagnosis (first-dementia ICD code date) to compute a measure of years until ADRD diagnosis. We also did not examine competing risk models incorporating the death of MVP participants, because of concerns of incompleteness of the reporting of death events for MVP participants. Lack of adjustment for competing risk, either because data on other censoring events are not available or because it is not modeled, can lead to inflated estimates of the rate of AD and the proportion of Veterans who would eventually develop dementia [75]. The magnitude of the estimated effects should therefore be interpreted with this caveat in mind.

Due to the strong correlation between PTSD and depression/anxiety symptoms (*r* = 0.77) in the MVP cohort and to avoid multicollinearity, we initially focused on analyses of PTSD, and did not also covary for depression in the multiple regression and survival models. We also examined interactions on an additive scale by calculating 95% confidence intervals (CIs) for the relative excess risk due to interaction (RERI) statistics. The RERI statistic reduces ambiguity when interpreting hazard ratios for significant multiplicative interactions in both logistic regression and Cox regression models (see [36, 42] for additional details). None of the additive-scale interactions from the Cox models were significant, hence we have not presented them here. Survival analysis results were further explored with the ‘*survminer’* package [33], and forest plots were created using the ‘*ggforestplot’* package by Nightingale Health. The first 10 ancestry principal components (PCs) were included as covariates in analyses to control for any cryptic population substructure. Within-ancestry PCs were calculated for each ancestry group using flashpca2.0 with the default settings based on 113,555 SNPs for EA, 170,207 SNPs for AA, and 116,435 SNPs for HA.

#### Demographic and lifestyle factors

Analyses also covaried for the following demographic and lifestyle factors: age at MVP Lifestyle Survey, gender, education, alcohol use, smoking history. Education was measured using a self-report item in the MVP Baseline Survey, which assessed education history on a 7-point scale ranging from “*Less than high school*” to “*Professional or Doctorate degree*.” Dichotomous yes/no smoking was coded based on self-report of > 100 cigarettes smoked (lifetime). Alcohol use was assessed using the AUDIT-C, a three-item measure of alcohol frequency, quantity, and binge drinking [14], which was included in the MVP Baseline Survey. A “heavy drinking” variable was created by dichotomizing AUDIT-C total score using the established clinical cut-off of >  = 4 for men and >  = 3 for women [13].

## Results

### Demographic, lifestyle, and clinical characteristics

Univariate demographic, lifestyle, and clinical descriptive statistics for each ancestry, stratified by age group, are displayed in Table 1. Due to the large sample size, results of ANOVA and chi-square tests contrasting means and proportional differences between age groups within each ancestry were significant for 29 of 30 tests. The patterns observed in the descriptive data across age groups were relatively similar for the EA, AA, and HA cohorts. There were differences in mean levels of SCC between ancestry groups, with AA and HA individuals having 1–2 points higher SCC on average than EA across the three age bins. Probable TBI was more prevalent in younger Veterans. PTSD and depression/anxiety symptoms were significantly lower (less severe) in older age groups compared to younger age groups, which is also consistent with prior literature on depression and anxiety disorders and aging [34, 64]. Perhaps surprisingly given our interest in the relationship between SCC and dementia, SCC was lower in the older rather than the younger age group. However, a post-hoc regression analysis including age, PTSD symptoms (PCL), and depression/anxiety symptoms (PHQ-4), indicated that lower PTSD and depression/anxiety in the older Veterans was likely driving this trend. After accounting for the effect of depression/anxiety and PTSD symptoms, age was positively associated with SCC (*p* = 91.07e-170; Supplemental Table 3).

**Table 1:** Demographic, lifestyle, and clinical characteristics of European, African, and Hispanic ancestry individuals stratified by age group

Variable	European Ancestry				African Ancestry				Hispanic Ancestry			
	**Age 45–54**	**Age 55–64**	**Age 65 + **	***p***	**Age 45–54**	**Age 55–64**	**Age 65 + **	***p***	**Age 45–54**	**Age 55–64**	**Age 65 + **	***p***
**N**	10,286	31,345	101,667		2,957	6,308	6,985		1,141	2,439	3,219	
**Age (mean/SD)**	50.75 (2.79)	61.27 (2.85)	73.66 (7.07)	*p* < 1E-250	50.91 (2.80)	60.36 (2.92)	71.66 (5.91)	*p* < 1E-250	50.53 (2.87)	60.86 (2.92)	71.94 (6.18)	*p* < 1E-250
**Male (n/%)**	8322 (80.90)	28,015 (89.38)	99,321 (97.69)	*p* < 1E-250	2,185 (73.89)	5,508 (87.32)	6,749 (96.62)	*p* < 1E-250	1,000 (83.89)	2,371 (92.04)	4,183 (97.76)	4.19E-95
**Smoking history (n/%)**	5,558 (54.03)	21,758 (69.41)	73,570 (72.36)	*p* < 1E-250	1,300 (43.96)	4,286 (67.95)	5,111 (73.17)	*p* < 1E-250	564 (47.32)	1680 (65.22)	3001 (70.13)	1.01E-73
**Heavy alcohol use (n/%)**	2,836 (27.57)	7,865 (25.09)	24,864 (23.47)	6.97E-53	747 (25.26)	1,411 (22.37)	1,164 (16.66)	6.16E-32	310 (26.01)	626 (24.30)	879 (20.54)	2.12E-14
**Education (mean/SD)**	3.95 (1.41)	3.65 (1.43)	3.72 (1.58)	*p* < 1E-250	3.74 (1.37)	3.39 (1.34)	3.42 (1.46)	6.67E-79	3.83 (1.35)	3.46 (1.33)	3.37 (1.44)	2.03E-168
**Depression/anxiety symptoms (mean/SD)**	3.16 (3.47)	2.57 (3.18)	1.45 (2.47)	*p* < 1E-250	3.58 (3.80)	3.02 (3.51)	2.14 (3.09)	3.12E-118	3.80 (3.73)	3.24 (3.52)	2.42 (3.25)	1.54E-57
**Probable TBI (n/%)**	1,397 (13.58)	3,438 (10.97)	7,281 (7.16)	9.84E-175	151 (5.11)	324 (5.14)	292 (4.18)	1.28E-7	158 (13.26)	191 (7.41)	291 (6.80)	1.53E-18
**PTSD symptoms (mean/SD)**	34.59 (16.18)	32.34 (15.00)	27.55 (12.51)	*p* < 1E-250	38.15 (18.70)	35.90 (17.50)	32.70 (16.35)	6.02E-71	39.27 (18.46)	37.20 (17.70)	34.02 (16.96)	8.01E-37
**APOE ε4 carrier (n/%)**	2,648 (26.75)	7,842 (25.36)	23,993 (23.94)	3.32E-21	1,169 (39.53)	2,355 (37.33)	2,479 (35.49)	1.65E-50	251 (21.06)	500 (19.41)	845 (19.75)	0.32
**Cognitive concerns (mean/SD)**	6.40 (7.11)	5.34 (6.63)	4.24 (5.87)	*p* < 1E-250	7.51 (8.07)	7.19 (8.04)	6.41 (7.49)	2.64E-17	8.01 (8.26)	6.96 (7.63)	6.53 (7.65)	6.81E-15
**ADRD (n/%)**	23 (0.22)	271 (0.86)	3597 (3.58)	1.52E-244	11 (0.39)	75 (1.26)	292 (4.56)	1.65E-50	2 (0.17)	26 (1.07)	197 (6.12)	1.70E-33

Analysis of variance, Chi-square tests, and Kruskal–Wallis (non-parametric) tests were used to examine the differences between age groups within each ancestry

*TBI* traumatic brain injury, *PTSD* Posttraumatic stress disorder, *ADRD* Alzheimer’s Disease and related dementias

### Associations between dementia risk factors and subjective cognitive concerns

**Fig. 1:**
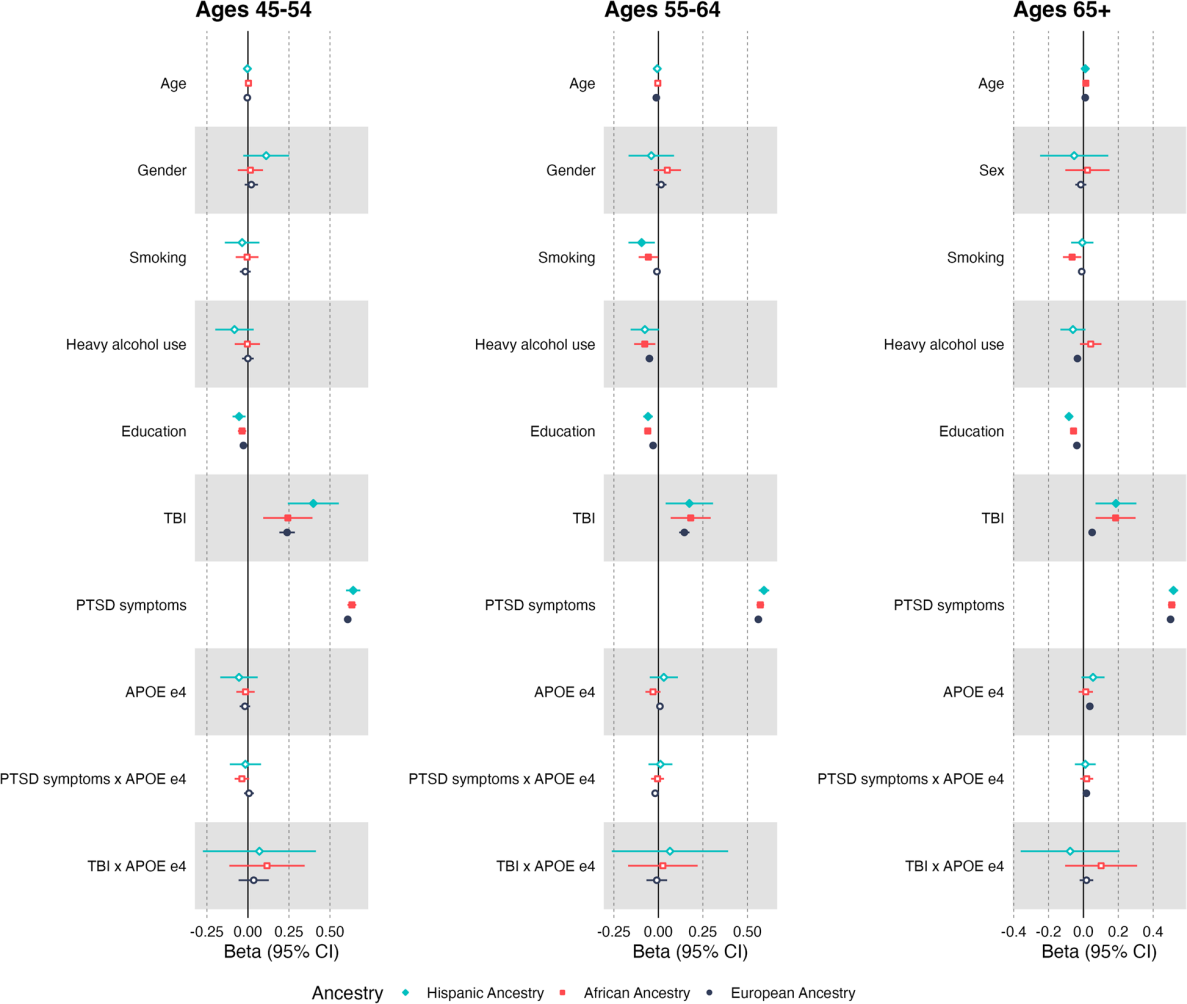
*Comparisons of Main and Interaction Effects Predicting Subjective Cognitive Concerns*. Filled point estimates indicate significant effects where *p*-values < 0.05. Open point estimates indicate non-significant effects. Sex was coded such that males = 0 and females = 1. TBI = traumatic brain injury, PTSD = posttraumatic stress disorder, SCC = subjective cognitive concerns

The results of the SCC regression models are presented in Table 2 and Fig. 1. In the EA cohort, we observed significant positive main effects of probable TBI and PTSD symptoms on SCC in all age groups. In the Age 65 + group, a significant main effect of *APOE* ε4 emerged, as well as a modest yet significant interaction between *APOE* ε4 and PTSD symptoms (*p* = 0.006). The nature of the interaction suggests that the association between PTSD severity and cognitive concerns was greater as a function of *APOE* ε4 in EA individuals age 65 + . However, given the small magnitude of the effect, this interaction is not likely to be clinically relevant. There was no evidence of an interaction between *APOE* ε4 and probable TBI on cognitive concerns in the EA cohort. Lower education was significantly associated with greater SCC in all EA age groups. Heavy alcohol use was negatively associated with SCC in the Age 55–64 and 65 + groups, suggesting problematic alcohol use was associated with fewer cognitive concerns. Female participants reported lower SCC relative to male participants in the EA Age 65 + group. Smoking history was not significantly associated with SCC in any EA age groups.

**Table 2:** Results of linear regression predicting subjective cognitive concerns

	**European Ancestry**				**African Ancestry**				**Hispanic Ancestry**			
	**Beta**	**(SE)**	**t-value**	***p-value***	**Beta**	**(SE)**	**t-value**	***p-value***	**Beta**	**(SE)**	**t-value**	***p-value***
**Age 45–54**	***n* = 10,286**				***n* = 2,957**				***n* = 1,227**			
Age	-0.004	(.003)	-1.332	0.183	0.003	(.006)	0.512	0.608	-0.003	(.009)	-0.357	0.721
Sex	0.021	(.021)	1.010	0.313	0.015	(.039)	0.384	0.701	0.111	(.071)	1.561	0.119
Smoking	-0.016	(.017)	-0.952	0.341	-0.005	(.035)	-0.144	0.886	-0.035	(.054)	-0.659	0.510
Heavy Alcohol Use	-0.000	(.018)	-0.017	0.986	-0.004	(.039)	-0.090	0.928	-0.082	(.060)	-1.371	0.171
Education	-0.027	(.006)	-4.537	5.78E-06	-0.036	(.013)	-2.775	0.006	-0.054	(.020)	-2.675	0.008
Probable TBI	0.239	(.024)	9.929	3.97E-23	0.243	(.077)	3.178	0.002	0.398	(.079)	5.022	5.89E-07
PTSD symptoms	0.608	(.008)	78.639	*p* < 1E-250	0.633	(.014)	46.323	*p* < 1E-250	0.641	(.022)	28.963	1.67E-140
*APOE* ε4	-0.018	(.016)	-1.128	0.259	-0.015	(.029)	-0.513	0.608	-0.054	(.058)	-0.935	0.350
*APOE* ε4 x PTSD symptoms	0.006	(.015)	0.428	0.669	-0.036	(.023)	-1.633	0.103	-0.015	(.048)	-0.312	0.755
*APOE* ε4 x TBI	0.035	(.047)	0.750	0.453	0.112	(.116)	0.969	0.333	0.073	(.175)	0.420	0.675
**Age 55–64**	***n* = 31,345**				***n* = 6,308**				***n* = 2,605**			
Age	-0.012	(.002)	-7.226	5.08E-13	-0.003	(.004)	-0.764	0.445	-0.006	(.006)	-1.041	0.298
Sex	0.016	(.015)	1.018	0.309	0.050	(.039)	1.278	0.201	-0.040	(.065)	-0.611	0.541
Smoking	-0.008	(.010)	-0.775	0.438	-0.056	(.028)	-2.017	0.044	-0.094	(.038)	-2.489	0.013
Heavy Alcohol Use	-0.050	(.011)	-4.740	2.15E-06	-0.077	(.030)	-2.526	0.012	-0.076	(.041)	-1.857	0.063
Education	-0.030	(.003)	-8.804	1.39E-18	-0.060	(.010)	-6.012	1.94E-09	-0.058	(.014)	-4.236	2.35E-05
Probable TBI	0.146	(.015)	9.830	9.00E-23	0.182	(.057)	3.168	0.002	0.174	(.068)	2.557	0.011
PTSD symptoms	0.562	(.005)	120.478	*p* < 1E-250	0.573	(.011)	52.805	*p* < 1E-250	0.593	(.015)	39.499	*p* < 1E-250
*APOE* ε4	0.009	(.009)	0.929	.353	-0.030	(.022)	-1.403	0.161	0.031	(.040)	0.757	0.449
*APOE* ε4 x PTSD symptoms	-0.018	(.009)	-1.543	0.055	-0.004	(.018)	-0.251	0.802	0.012	(.034)	0.337	0.736
*APOE* ε4 x TBI	-0.009	(.030)	-0.301	0.763	0.029	(.101)	0.285	0.776	0.005	(.164)	0.028	0.978
**Age 65 + **	***n* = 101,667**				***n* = 6,985**				***n* = 4,360**			
Age	0.010	(.000)	29.361	1.08E-188	0.014	(.002)	6.789	1.22E-11	0.011	(.002)	4.252	2.17E-05
Sex	-0.015	(.016)	-0.939	0.348	0.023	(.065)	0.355	0.723	-0.053	(.100)	-0.532	0.595
Smoking	-0.010	(.006)	-1.763	0.078	-0.065	(.027)	-2.431	0.015	-0.007	(.033)	-0.200	0.842
Heavy Alcohol Use	-0.034	(.006)	-6.027	1.68E-09	0.042	(.031)	1.330	0.184	-0.060	(.037)	-1.639	0.101
Education	-0.038	(.002)	-23.683	1.18E-123	-0.057	(.008)	-6.965	3.58E-12	-0.083	(.010)	-7.923	2.94E-15
Probable TBI	0.050	(.010)	5.252	1.51E-07	0.185	(.058)	3.157	0.002	0.187	(.060)	3.117	0.002
PTSD symptoms	0.500	(.003)	165.001	*p* < 1E-250	0.507	(.011)	46.066	*p* < 1E-250	0.517	(.014)	37.840	*p* < 1E-250
*APOE* ε4	0.037	(.005)	7.044	1.88E-12	0.013	(.021)	0.634	0.526	0.055	(.034)	1.632	0.103
PTSD symptoms x *APOE* ε4	0.017	(.006)	2.741	0.006	0.019	(.019)	0.999	0.318	0.011	(.030)	0.351	0.725
TBI x *APOE* ε4	0.017	(.020)	0.877	0.381	0.102	(.106)	0.957	0.339	-0.045	(.144)	-0.309	0.757

The parameter estimates for the main effects listed were derived from main effects-only models. The interaction term parameter estimates were from models with the main effects and interaction term included in the same model. Sex was coded such that males = 0 and females = 1

*TBI* traumatic brain injury, *PTSD* posttraumatic stress disorder

In the AA cohort, we observed significant positive main effects of probable TBI and PTSD symptom severity on SCC across age groups. There was no evidence of a main effect of *APOE* ε4 or interactions between PTSD symptoms or probable TBI and *APOE* ε4 on SCC. In the AA Age 55–64 cohort, there was a significant negative association between heavy alcohol use and SCC, but this was not observed in other age groups. There was also a significant negative effect of smoking history on SCC in the 55–64 and 65 + groups, suggesting lifetime smokers reported lower cognitive concerns. Lower education was associated with greater SCC in all AA age groups.

In the HA cohort, we also observed significant positive main effects of TBI and PTSD symptom severity on SCC across all age groups. There was no evidence of a main effect of *APOE* ε4 or interactions between PTSD symptoms or TBI and *APOE* ε4. We also found that lower education was associated with higher SCC in all HA age groups.

### Survival analysis: risk for ADRD as a function of PTSD, probable TBI, and SCC

**Fig. 2:**
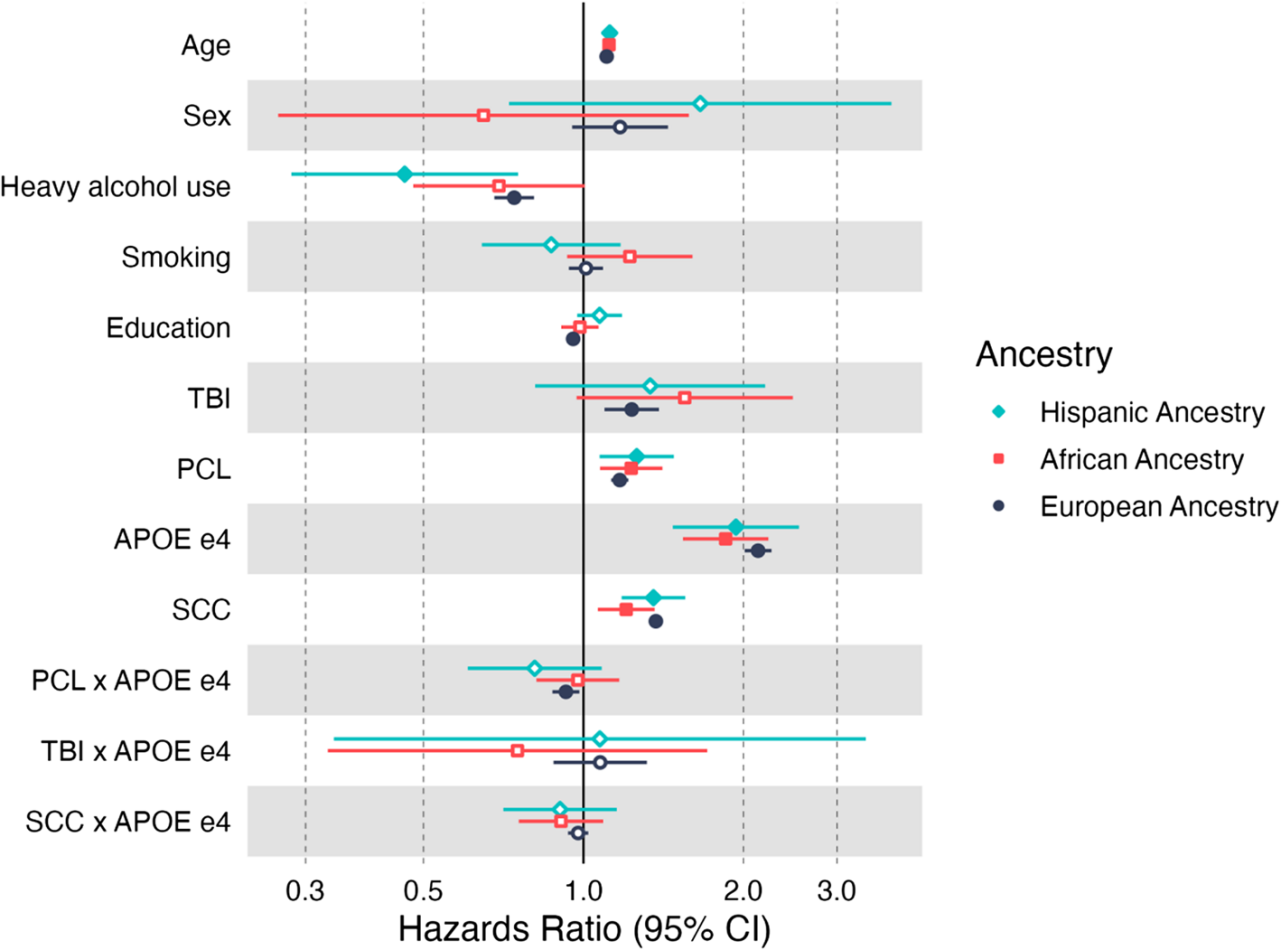
*Comparison of ADRD Hazard Ratios in European, African, and Hispanic Ancestry Individuals Age 65 +* . Filled point estimates indicate significant effects where *p*-values < 0.05. Open point estimates indicate non-significant effects. Sex was coded such that males = 0 and females = 1. TBI = traumatic brain injury, PTSD = Posttraumatic stress disorder, ADRD = Alzheimer’s Disease and related dementias, SCC = subjective cognitive concerns

**Fig. 3:**
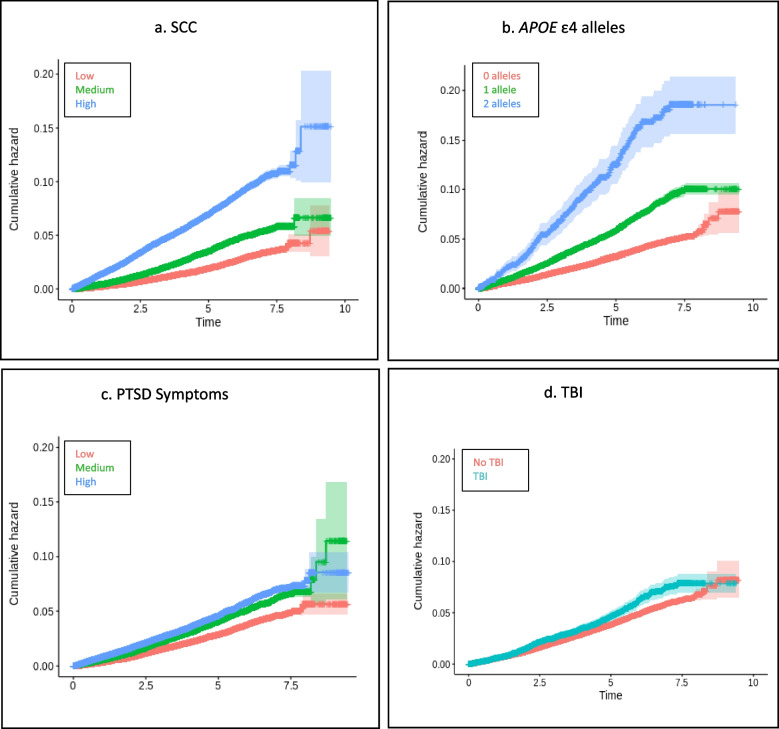
**a**-**d** *Survival Curves for Veterans of European Ancestry Age 65 and Older*. Panels show Kaplan–Meier survival curves demonstrating the relationship between SCC, APOE ε4, PTSD symptoms, TBI, and incidence of ADRD in Veterans of European ancestry age 65 and older. TBI = traumatic brain injury, PTSD = posttraumatic stress disorder, ADRD = Alzheimer’s Disease and related dementias, SCC = subjective cognitive concerns

**Fig. 4:**
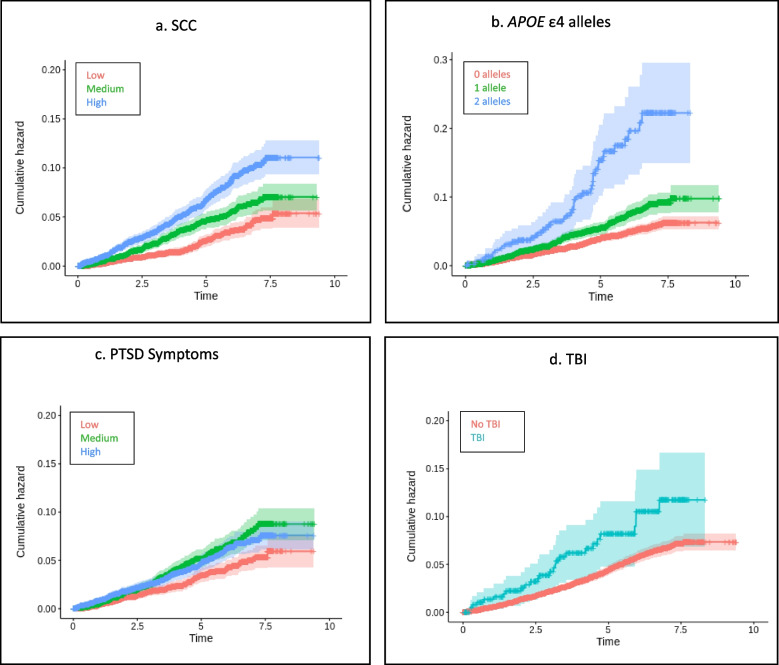
**a**-**d** S*urvival Curves for Veterans of African Ancestry Age 65 and Older.* Panels show Kaplan–Meier survival curves demonstrating the relationship between SCC, APOE ε4, PTSD symptoms, TBI, and incidence of ADRD in Veterans of African ancestry age 65 and older. TBI = traumatic brain injury, PTSD = Posttraumatic stress disorder, ADRD = Alzheimer’s Disease and related dementias, SCC = Subjective Cognitive Concerns

**Fig. 5:**
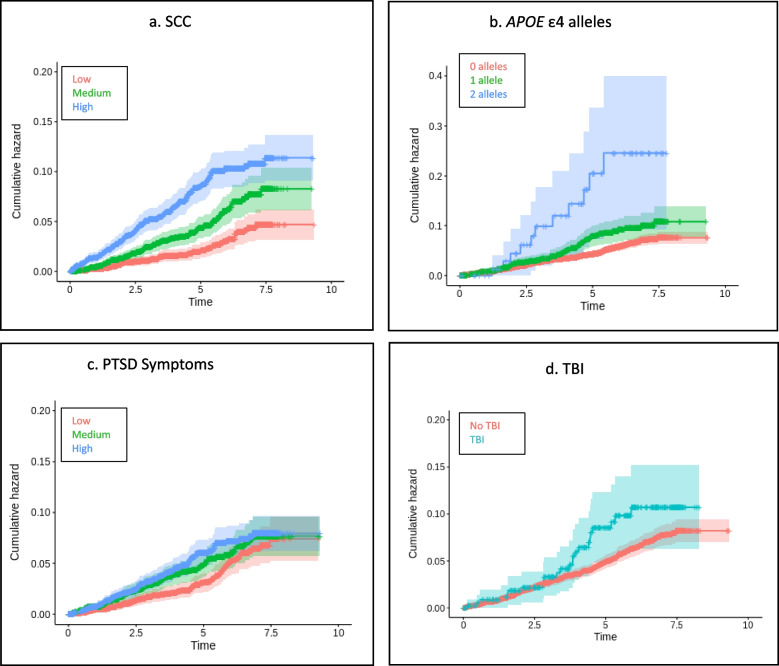
**a**-**d** *Survival Curves for Veterans of Hispanic Ancestry Age 65 and Older*. Panels show Kaplan–Meier survival curves demonstrating the relationship between SCC, APOE ε4, PTSD symptoms, TBI, and incidence of ADRD in Veterans of Hispanic ancestry age 65 and older. TBI = traumatic brain injury, PTSD = Posttraumatic stress disorder, ADRD = Alzheimer’s Disease and related dementias, SCC = Subjective Cognitive Concerns

Results of the Cox proportional hazards models for each ancestry group are presented in Table 3. The number of ADRD cases, average time to ADRD diagnosis, and average censoring period across the ancestry groups are provided in Table 4. A summary of the HR estimates and 95% CIs for our primary variables of interest and covariates for all three ancestry groups is provided in the forest plot in Fig. 2. SCC, older age at enrollment, PTSD symptoms, and *APOE* ε4 were associated with increased rates of ADRD in all three ancestry groups. Heavy alcohol use was significantly associated with rate of ADRD in the EA and HA ancestry groups. Education and probable TBI were only associated with rate of ADRD in the EA group. However, the estimated effect of direction was the same across cohorts, and probable TBI approached significance in the AA cohort. Figures 3, 4 and 5 display the univariate relative risk for ADRD across time as a function of our variables of interest: SCC, *APOE* ε4, PTSD symptoms, and TBI in the EA, AA, and HA cohorts respectively.

**Table 3:** Results of Cox regression models predicting EMR-determined ADRD diagnoses

Variable	European Ancestry *n* = 90,548				African Ancestry *n* = 6,254				Hispanic Ancestry *n* = 3,861			
		95.0% Cl				95.0% Cl				95.0% Cl		
	HR	Lower	Upper	*p*	HR	Lower	Upper	*p*	HR	Lower	Upper	*p*
**Age**	1.11	1.10	1.11	*p* < 1e-250	1.12	1.10	1.14	1.37e-39	1.12	1.10	1.14	1.69E-30
**Sex**	1.17	0.95	1.44	0.134	0.65	0.27	1.58	0.340	1.66	0.72	3.80	0.231
**Heavy alcohol use**	0.74	0.68	0.81	7.88e-12	0.69	0.48	1.01	0.053	0.46	0.28	0.75	0.002
**Smoking**	1.01	0.94	1.09	0.775	1.22	0.93	1.60	0.147	0.87	0.64	1.17	0.362
**Education**	0.96	0.94	0.98	3.88e-05	0.98	0.91	1.07	0.710	1.07	0.97	1.18	0.156
**Probable TBI**	1.23	1.10	1.39	5.08e-04	1.55	0.97	2.48	0.066	1.34	0.81	2.20	0.256
**PTSD symptoms**	1.17	1.13	1.21	8.18e-17	1.23	1.07	1.41	0.003	1.26	1.07	1.48	0.005
***APOE* ε4**	2.13	2.01	2.26	5.25e-143	1.85	1.54	2.23	6.89e-11	1.94	1.47	2.54	2.16E-06
**SCC**	1.37	1.33	1.41	2.37e-114	1.20	1.06	1.36	0.003	1.35	1.18	1.55	1.62E-05
**PTSD symptoms x *APOE* ε4**	0.93	0.87	0.98	0.01	0.98	0.82	1.17	0.791	0.81	0.61	1.08	0.154
**TBI x *APOE* ε4**	1.08	0.88	1.32	0.482	0.75	0.33	1.71	0.496	1.07	0.34	3.40	0.904
**SCC x *APOE* ε4**	0.98	0.94	1.02	0.302	0.91	0.76	1.09	0.297	0.90	0.71	1.15	0.418

Hazard ratio (HR) greater than one indicate elevated proportional risk for ADRD, and below one indicates reduced proportional risk for ADRD. The parameter estimates for the main effects listed were derived from main effects-only models. The interaction term parameter estimates were from models with the main effects and interaction term included in the same model. Analyses controlled for ancestry-specific principal components. Sex was coded such that males = 0 and females = 1

*TBI* traumatic brain injury, *PTSD* posttraumatic stress disorder, *ADRD* Alzheimer’s Disease and related dementias, *SCC* Subjective Cognitive Concerns

**Table 4:** Prevalence of ADRD diagnosis, years to ADRD diagnosis, and years censored

Variable	European Ancestry *n* = 90,548	African Ancestry *n* = 6,254	Hispanic Ancestry *n* = 3,861
ADRD diagnosis (N (%))	4,106 (1.91)	385 (2.41)	234 (2.91)
Years to ADRD Diagnosis (Mean (SD))	3.34 (2.91)	3.50 (1.86)	3.34 (1.92)
Years Censored (Mean (SD))	5.36 (1.93)	5.38 (1.91)	5.38 (1.88)

*ADRD* Alzheimer’s Disease and related dementias

## Discussion

In this study, we examined associations between established dementia risk factors (PTSD, TBI, and *APOE* ε4) and SCC, and the prognostic value of SCC in relationship to future EMR-determined ADRD diagnoses. This was evaluated in large cohorts of European, African, and Hispanic ancestry middle- and old-aged U.S. Veteran participants in MVP, one of the world’s largest biobanks. Our results confirm the previously reported strong link between SCC and PTSD (see e.g. [16, 25, 47, 52, 66]). However, our findings also suggest that SCC is related to ADRD biological risk, as evident in the association between SCC and *APOE* ε4 among older adults of European ancestry. Results further indicated that SCC was predictive of future EMR-determined ADRD diagnoses across all ancestry groups evaluated. This highlights the challenges of interpreting SCC as it is sensitive to true ADRD biological risk and may signal incipient ADRD but is also a reflection of psychological symptoms. The relationship between EMR-determined ADRD diagnoses and dementia risk factors such as PTSD and TBI was largely consistent with our prior cross-sectional research both in the MVP EA and AA ancestry cohorts [42] and in the broader literature. However, the Cox models used here offer substantial advantages relative to the cross-sectional logistic-regression-based framework in that it explicitly models time to ADRD diagnosis. This makes the Cox model more suitable for capturing inter-individual variability in disease onset and provides more accurate risk assessment.

Consistent with the concept of SCC as a prodromal dementia indicator, SCC was associated with increased risk for EMR-derived ADRD in older Veterans across all ancestry groups, in models incorporating the effects of competing risk factors such as PTSD symptoms and probable TBI, all of which were also associated with increased rate of ADRD. Current guidance on screening for MCI and dementia advises against relying only on self-reported cognitive complaints, due to risk of diagnostic imprecision [54]. However, our results suggest that SCC should not be disregarded, even in older Veterans with symptoms of PTSD or history of TBI. SCC noted by patients or caregivers should be followed up with further evaluation using a validated neurocognitive assessment tool, and referral to providers who specialize in neurocognitive symptoms as indicated [54, 69].

Our investigation of the predictors of SCC in the middle-aged and older-aged Veteran cohorts further highlights the significance of SCC for dementia. In older EA Veterans who reported lower levels of PTSD and depression symptomatology, we observed an association between *APOE ε4* and SCC. Thus, although the association between SCC and PTSD and TBI symptomatology is robust, *APOE*-associated neuropathology is also likely contributing to SCC in this group. Parallel to genetic effects on dementia, prior research has indicated that the magnitude of genetic effects on subjective concerns increases over time [11] and again suggests that SCC should not be ignored or dismissed. The *APOE ε4* association with SCC was only observed in the EA Veterans in this study and was not evident in the smaller AA and HA cohorts. This is likely due to lower statistical power in these smaller cohorts due to sample size in combination with known differential effects of *APOE* ε4 by ancestry [19, 70, 71].

We examined MVP participants (EA, AA, and HA) stratified by ancestry for several reasons. First, stratification can accommodate the known differences in *APOE* ε4 effects across ancestry groups and improve the representation of AA and HA Veterans in genetics research. It is also important to look for potential differences by ancestry for non-genetic ADRD risk factors (e.g., cardiometabolic health, healthcare access, socioeconomic and neighborhood factors; [7]) as rates of these risk factors also differ by ancestry in the US and can complicate the interpretation of differential associations between SCC and ADRD. We examined education, PTSD, heavy alcohol use, and cigarette use and found lower prevalence of heavy alcohol use but greater PTSD symptom severity among AA and HA cohorts relative to the EA cohorts. SCC was also higher in the AA and HA cohorts. Yet, when we looked at the relationship between these demographic and environmental exposure factors and SCC, we did not find convincing evidence that these associations differed by ancestry. Similarly, in the ADRD risk models, we observed comparable effect size estimates for the three ancestry groups, albeit some associations were only statistically significant in the EA group due to the reduced sample size in the AA and HA cohorts. The 95% CIs for the hazard ratios overlapped across ancestry cohorts for all demographic and environmental factors, suggesting no differential effect of SCC, PTSD, or TBI on ADRD risk across ancestries. Overall, these results are consistent with well-known differences in the prevalence of psychiatric disorders and adverse health factors in AA versus EA Americans, such as differences in education [9, 10, 22], and do not appear to suggest differential effects of these factors on ADRD risk. This mirrors earlier work which found that the impact of education, head injury, and alcohol use on AD risk was similar in EA and AA families [8]. The lack of differential association across ancestries carries important clinical implications given documented healthcare disparities in ADRD care across race and ethnicity. Surveys of non-White US adults and dementia caregivers have indicated that their race and ethnicity can present a barrier for receiving adequate treatment and that staff and providers do not listen to their concerns [2]. It is critical that healthcare providers not dismiss SCC in minoritized populations or assume SCC is better accounted for by other demographic, psychological, or environmental factors as this may delay access to needed dementia care, as has been demonstrated in other health conditions such as cardiac care [63]. Rather, healthcare providers should take reports of SCC seriously and refer individuals for further evaluation and monitoring in order to avoid contributing to a widening gap in healthcare access and disease outcomes.

## Limitations

The findings from this study should be interpreted in the context of several limitations. First, our survival analysis focused on an ICD-derived ADRD classification. This was in part due to the lack of available biomarker and neurocognitive test data in the EMR which would allow us to accurately differentiate AD from other forms of dementia. Because of this, the estimated *APOE* ε4 effect size is somewhat lower than estimates obtained from studies relying on neurocognitive tests or associated biomarkers. We also did not have a measure of SCD in our sample, that is, a sense of having decreased cognitive ability or greater difficulty than one has had in the past. Objective measures of cognitive functions and SCD may show a different pattern of association with demographic factors and ADRD risk than SCC [31]. Second, we note that the interpretation of a link between *APOE* ε4 and SCC as an underlying expression of AD pathology in those 65 + is predicated to some degree on the participants being unaware of their *APOE* genotype. That is, if knowledge of their *APOE* genotype was prevalent among MVP participants, it is quite possible that this knowledge might influence their SCC. However, the subsequent association of SCC with risk of ADRD in models which included *APOE* ε4 as a covariate further reinforces the notion that SCC is at least partially influenced by underlying AD pathology, and that knowledge of *APOE* genotype is not a major confounder in this case. If *APOE* testing becomes more widespread, either through its increased use as part of medical care or through widespread direct-to-consumer genetic testing (e.g., as performed by 23andMe), the associations observed here could change. In future studies of subjective cognitive and memory concerns and/or decline, it will be important to investigate the impact of knowledge of *APOE* genotype along with the *APOE* genotype itself. Third, analyses did not account for competing risk of death, due to limitations in the availability of death data at the time of the MVP 20.1 phenotype release. As a result, our Cox models likely overestimate risk relative to the estimates that would be obtained using a competing risk model with full data on deaths of MVP participants [60, 78]. Competing risk analyses represent an important next step for this research and would be necessary to obtain more precise estimates of the rate of ADRD in Veterans at risk. Finally, we note that our results are based on a large sample of primarily male US Veterans. While these findings may not generalize to the civilian population, the inclusion of African- and Hispanic-ancestry individuals in this research may help to address the underrepresentation of minority groups in ADRD genomic research and the broader ADRD literature [40, 58].

## Conclusions

This study addressed two important questions concerning whether the relationship between established genetic and environmental risk factors for EMR-derived ADRD diagnoses could also be observed in SCC across varying ages, and if SCC, alone or in combination with other ADRD risk factors, was associated with future EMR-determined ADRD diagnosis. These questions were evaluated in large samples of EA, AA, and HA U.S. military Veterans. Our results demonstrated the significance of the interplay between psychiatric symptoms (PTSD), AD genetic risk as measured by *APOE* ε4, and probable TBI, in predicting SCC and subsequent risk for ADRD diagnosis. The findings underscore the value of SCC as an indicator of ADRD risk in individuals 65 and older when considered in conjunction with other influential genetic and environmental risk factors. Importantly, we emphasize the need for careful evaluation, monitoring, and early intervention to delay ADRD onset or slow its progression, given the nuanced relationship between cognitive concerns, psychopathology, and genetic predisposition in older adulthood. The current research not only advances our understanding of ADRD risk prediction but also highlights the importance of addressing both psychiatric symptoms and biological drivers of the disease. As precision medicine evolves, these insights call for a well-rounded approach to ADRD prevention and treatment, considering psychiatric symptoms as well as genetic and other biological vulnerabilities. This study also contributes to the broader efforts to characterize ADRD pathology in Veterans, particularly in the understudied African- and Hispanic-ancestry populations. We hope this will ultimately guide more equitable and effective strategies for early detection and intervention of ADRD.

### Supplementary Information

Supplementary Material 1.

## Data Availability

The data, code, and phenotypes underlying this publication is accessible to researchers with MVP data access. Due to VA policy, MVP is currently only accessible to researchers VA-funded MVP project, either through a VA Merit Award or a career development award. See https://www.research.va.gov/funding/Guidance-MVP-Data-Access-Merit-Award.pdf for MVP access details.

## Abbreviations

AA: African Ancestry
AD: Alzheimer’s Disease
ADRD: Alzheimer’s Disease and Related Dementias
*APOE* ε4: Apolipoprotein E isoform 4
EA: European Ancestry
EMR: Electronic Medical Record
HA: Hispanic Ancestry
ICD: International Classification of Diseases
MCI: Mild cognitive impairment
MVP: Million Veteran Program
PCs: Principal Components
PTSD: Posttraumatic stress disorder
SCC: Subjective cognitive concerns
SCD: Subjective cognitive decline
TBI: Traumatic brain injury
VA: United States Department of Veterans Affairs
