# Supplementary material for: Subjective cognitive concerns, APOE ε4, PTSD symptoms, and risk for dementia among older veterans
Source: Alzheimers Res Ther. 2024 Jun 29;16:143. doi: 10.1186/s13195-024-01512-w (PMC11218206; doi:10.1186/s13195-024-01512-w)
Supplement: Supplementary file 1 — Supplementary Material 1. [file 13195_2024_1512_MOESM1_ESM.docx]

| **Supplemental Table 1.**  ICD Codes used to identify ADRD cases and to exclude controls. |
| --- |
| **ADRD CD 9 Codes:** |
| Alzheimer’s disease 331.0 |
| Unspecified dementia without behavioral disturbance 294.20 |
| Unspecified dementia with behavioral disturbance 294.21 |
| Other persistent mental disorders/Other specified organic brain syndromes 294.8 |
| Senile dementia uncomplicated 290.0 |
| Senile dementia with delusional features 290.20 |
| Senile dementia with depressive features 290.21 |
| Senile dementia with delirium 290.3 |
| **ADRD ICD 10 codes:** |
| Alzheimer’s disease G30.1, 30.8, and 30.9 |
| Unspecified dementia without behavioral disturbance F03.90 |
| Unspecified dementia with behavioral disturbance F03.91 |
| Vascular dementia: 290.40, 290.41, 290.42, 290.43, F01.50, F01.51 |
| Lewy Body dementia: 331.82; G31.83 |
| Frontotemporal/Other Fronto Dementia: 331.1, G31.0; 331.19, G31.09 |
| Presenile dementia: 290.10, 290.11, 290.12, 290.13. |
| **Other Dementia Codes:** |
| Huntington’s Disease: 333.4; G10 |
| Parkinson’s Disease: 332; G20. |
| Creutzfeldt-Jakob Disease: A81.00. |
| Pick’s Disease of the Brain: 331.11, G31.01 |
| Korsakoff Syndrome: F10.96. |
| Idiopathic Normal Pressure Hydrocephalus: 331.5, G91.2 |
| Dementia in conditions classified elsewhere W/WO behavioral        disturbance: 294.10/294.11 |
| Dementia in other diseases classified elsewhere W/WO behavioral disturbance:        F02.80, F02.81 |
| **MCI codes:** |
| ICD 9: 331.83; ICD10: G31.84 |
| *Note:* ADRD cases were defined as individuals who received at least two ADRD ICD codes. Controls had no ADRD ICD codes, Other Dementia codes, Mild Cognitive Impairment codes, or prescription history of ADRD medications listed in Supplemental Table 2. |

**Supplemental Table 2.**

Prescription Medications for ADRD Used to Rule Out Control Participants

| **Medication Type** | **Medication Names** |
| --- | --- |
| Anti-amyloid antibody therapies | Aduhelm^®^ (aducanumab)  Leqembi^®^ (Lecanemab) |
| Cholinesterase inhibitors | Razadyne (galantamine)  Reminyl^®^ (galantamine)  Exelon^®^ (rivastigmine)  Aricept^®^ (donepezil)  Cognex^®^ (tacrine) |
| N-methyl-D-asparate receptor antagonists | Namenda ^®^ (memantine) |
| Cholinesterase inhibitor + glutamate regulators | Namzaric ^®^ (memantine + donepezil) |
| *Note:* Generic names are provided in parentheses | |

**Supplemental Table 3.**

Linear Regression Predicting Cognitive Symptoms in European Ancestry Aged 65 and Older.

| **Variable** | **Estimate (SE)** | **t-value** | **p-value** |
| --- | --- | --- | --- |
|  |  |  |  |
| Depression/anxiety symptoms | 0.08 (0.00) | 54.62 | *p<*1E-250 |
| PTSD symptoms | 0.34 (0.00) | 77.30 | *p<*1E-250 |
| Age | 0.01 (0.00) | 27.88 | 1.07E-170 |
| Model summary: *R^2^*=0.245, F(3, 98,801) = 1.069E+4, *p<*1E-250 | | | |
